# A cis-regulatory logic simulator

**DOI:** 10.1186/1471-2105-8-272

**Published:** 2007-07-27

**Authors:** Robert D Zeigler, Jason Gertz, Barak A Cohen

**Affiliations:** 1 Department of Genetics, Washington University School of Medicine, 4444 Forest Park Parkway, St. Louis, MO 63108, USA

## Abstract

**Background:**

A major goal of computational studies of gene regulation is to accurately predict the expression of genes based on the cis-regulatory content of their promoters. The development of computational methods to decode the interactions among cis-regulatory elements has been slow, in part, because it is difficult to know, without extensive experimental validation, whether a particular method identifies the correct cis-regulatory interactions that underlie a given set of expression data. There is an urgent need for test expression data in which the interactions among cis-regulatory sites that produce the data are known. The ability to rapidly generate such data sets would facilitate the development and comparison of computational methods that predict gene expression patterns from promoter sequence.

**Results:**

We developed a gene expression simulator which generates expression data using user-defined interactions between cis-regulatory sites. The simulator can incorporate additive, cooperative, competitive, and synergistic interactions between regulatory elements. Constraints on the spacing, distance, and orientation of regulatory elements and their interactions may also be defined and Gaussian noise can be added to the expression values. The simulator allows for a data transformation that simulates the sigmoid shape of expression levels from real promoters. We found good agreement between sets of simulated promoters and predicted regulatory modules from real expression data. We present several data sets that may be useful for testing new methodologies for predicting gene expression from promoter sequence.

**Conclusion:**

We developed a flexible gene expression simulator that rapidly generates large numbers of simulated promoters and their corresponding transcriptional output based on specified interactions between cis-regulatory sites. When appropriate rule sets are used, the data generated by our simulator faithfully reproduces experimentally derived data sets. We anticipate that using simulated gene expression data sets will facilitate the direct comparison of computational strategies to predict gene expression from promoter sequence. The source code is available online and as additional material. The test sets are available as additional material.

## Background

Transcriptional regulation of genes is controlled largely through the concerted action of combinations of cis-regulatory sites in the promoters and surrounding regulatory DNA of genes. The interactions between cis-regulatory sites can be complex and may include synergistic [1], competitive [2], and amplifying [3] interactions, and are often influenced by the spacing and orientation of the sites relative to each other and to the transcriptional start site [4,5]. The complexity of the "cis-regulatory code" makes predicting gene expression from promoter sequence a challenging problem.

Computational approaches for determining the cis-regulatory code include multiple regression models [6], Bayesian networks [7], logic operators [8], and machine learning methods [9]. Though their mathematical frameworks differ, all of these approaches use large-scale transcriptional data (usually microarray-based expression profiling data) and attempt to correlate expression patterns with the presence or absence of computationally predicted cis-regulatory motifs. Currently, we do not have good ways to compare the performance of these different approaches to each other or to new approaches being developed. A serious problem in comparing these methods is the lack of robust test data in which the cis-regulatory interactions underlying the expression data are accurately known. We need data in which the "true" answer is known if we are to compare methodologies. To address this limitation, we built a rule based simulator to create test data sets.

Simulators are playing a useful role in reconstructing gene regulatory networks (GRN). A GRN models the regulatory connections between genes, as opposed to the interactions between cis-regulatory sites in a promoter. Because the true GRN of a cell is not known, artificially created GRNs are used to evaluate the accuracy of algorithms that attempt to determine network architecture and dynamics [10]. GRN simulators provide test datasets [11,12], which in turn are used to assess the performance of network reconstruction techniques [13]. We anticipate that gene expression simulators will play a similar role in the development of computational approaches to decipher the interactions between cis-regulatory sites.

We present a regulatory rule simulator that generates random promoters and produces expression data based on user-defined interactions between cis-elements. Whereas a GRN simulator attempts to create a web of genes connected in a biologically relevant manner, our simulator generates promoter regions and predicts the expression from those promoters. We also present test datasets, created by the simulator, which can be used to assess the performance of algorithms that attempt to determine underlying regulatory rules. The promoter generator and simulator, named ReLoS (cis-**Re**gulatory **Lo**gic **S**imulator), are available for download [14] (see additional files 4 and 5). A web interface [15] is also available. The test data sets are available in additional file 1.

## Results and Discussion

### Simulating regulatory rules

**Figure 1:**
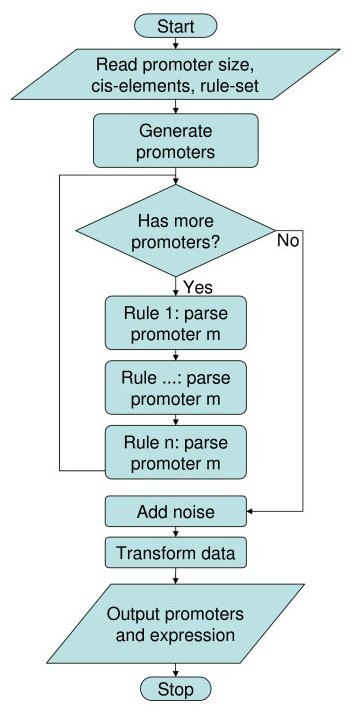
**Flow of Relos**. Users supply Relos with cis-elements to use, the number and size of promoters to generate, and the rules used to analyze the promoters. Relos generates the promoters then analyzes the rules by passing the promoters through a rule-pipeline of the user-defined rules. Noise is then added, and the data is optionally transformed via a sigmoidal transform to ensure upper and lower limits of expression.

Gene expression simulations using Relos are divided into discrete steps (Figure 1). The user first specifies the number of cis-regulatory sites that will be part of the simulation. Next, the user creates a rule set that defines the interactions between cis-regulatory sites and their effects on gene expression. Relos then generates a set of promoters consisting of random combinations of these cis-regulatory sites. Finally, the expression of each promoter is determined by applying the rule set to each promoter sequence. The simulator outputs a list of promoter sequences with their corresponding expression values. At every step, the user may specify parameters to customize the simulations

With Relos a user can encode a wide variety of cis-regulatory rules. The rules are defined in an XML simulation file to make the attributes of the simulation, including the rules, legible to the user. A single rule in a rule-set is defined by the cis-regulatory sites involved, the conditions required by the rule, conditions excluded by the rule, context dependencies for each condition, and the output expression generated by that rule. Logical relationships such as OR, NOT and AND can be expressed in describing interactions between sites. Constraints on the spacing, orientation, and distance of sites from each other can be incorporated into any rule. Rule outputs may be combined in linear and non-linear ways (see Methods). A rule may simply specify the additive contribution of a particular regulatory element, or it may determine the parameters of an epistatic (eg: cooperative, competitive, synergistic, etc.) interaction between elements. Promoters are parsed by each rule in the order in which the rules are specified. When a rule matches a promoter, however, that rule may specify a set of rules which should be skipped in the analysis of the matched promoter.

Promoter processing by rules is delegated to the "analyzer". The analyzer is responsible for determining whether a rule will affect a promoter, based on the constraints specified for the rule. The analyzer is also responsible for specifying the effect of a rule on the expression of a promoter. Analyzers serve as the central point of extensibility in Relos. For each rule, it is possible to specify a custom analyzer. Relos comes with a regular expression analyzer, which modifies promoter expression if the regular expression is matched. Another analyzer allows user-defined mathematical functions to be used to determine rule outputs. For example, a Hill function [16,17] might be used to describe cooperativity between sites. The flexibility inherent in the design of Relos allows users to simulate virtually any mode of regulation among cis-regulatory sites.

Real expression data are bounded. At the lower bound, a cell cannot express less than zero copies of a gene. There is also an upper limit of detection in any experimental setup and to the levels of RNA that can be produced when a promoter is fully occupied by the transcriptional machinery and transcribing at the maximum rate. These constraints produce sigmoid expression patterns. For this reason, Relos allows users to sigmoidally transform the output data. Users may explicitly tell Relos to transform the data. In this case, Relos uses a sigmoid transformation centered on the average expression for the simulation (see methods). Using the simulation expression mean to center the transformation allows rule-sets to be compared in terms of the variation present in the parsed promoters. Simulations with large variance will show a spread of values between zero and one. Simulations with little variance will, when transformed, cluster around the value of 0.5. One consequence of the mean-dependent transformation is that it is impossible to generate a transformed dataset in which all expression is either "on" or "off" since datasets with very little variation will result in midline expression when transformed. Users may therefore specify a rule at the end of the pipeline employing a custom analyzer to transform the data. Relos comes with a SigmoidalTransform analyzer (see Methods) that can be used for this purpose, but users may also provide their own transformations. The SigmoidalTransform analyzer uses four parameters (see Methods) to adjust the shape and scale of the transformation. These parameters are independent of the simulation dataset and determine an absolute scale of expression onto which all rule-sets are mapped. By using a consistent set of parameters, users can compare rule-sets with regard to their strength of expression and compare variances according to where the mean lies in the absolute expression scale. Since this transformation does not depend on the dataset, the absolute scale is arbitrarily determined by the choice of parameters and users should be careful to use rules consistent with the scale determined by the parameters.

In addition to rules, their analyzers and constraints, and transformation parameters, the XML simulation file contains other adjustable attributes for the simulation. For example, after the promoters have been interpreted using the current rule set, Gaussian noise is added by the simulator with a user defined standard deviation. Relos is also capable of generating random promoters based on user-defined properties, such as promoter length, cis-regulatory elements and their frequencies and outputting promoters in either fasta or Relos format. These synthetic promoters can be used directly by the simulator. For more details, see Methods.

**Figure 2:**
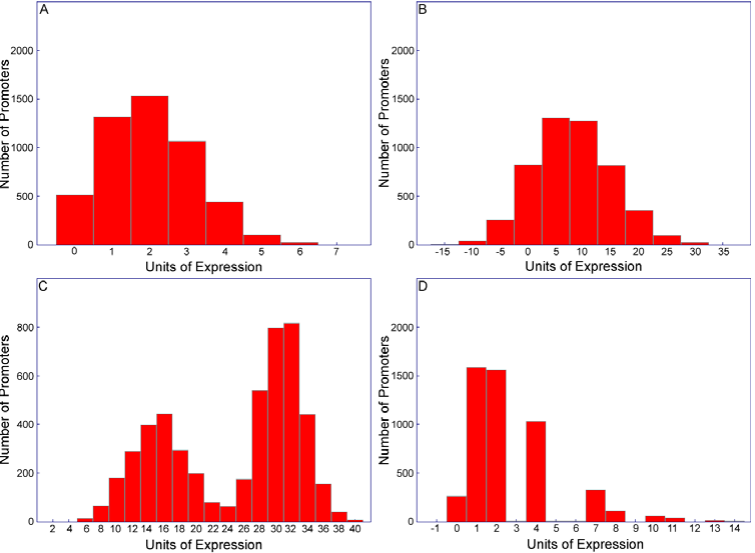
**Sample Relos outputs**. Relos was used to generate and analyze promoters using four different models. Five thousand promoters were generated in all Figure 2 simulations. ***A***. A simulation that depicts a single activator, modeled as an additive rule. ***B***. A simulation that depicts an activator and a repressor modeled as additive rules. ***C***. A simulation that depicts a synergistic rule between two regulatory elements. Each element has a small additive contribution to expression, but promoters with at least one of each element have enhanced expression. Gaussian noise was added to the output of the simulation at 5% of the level of expression of individual promoters. ***D***. A simulation that depicts a cooperative interaction between two regulatory elements modeled with a hill function. Noise was added to the simulation as in *C*.

Examples of simulated datasets are shown in Figure 2. As a visual aid to interpret the output of the simulations, histograms illustrating the distribution of expression values are shown. Figure 2a shows the distribution of expression values for 5000 fixed-length random promoters consisting of variable numbers of a single type of cis-regulatory activator site and neutral spacer elements, where all elements are equally probable. The expression is therefore a reflection of the distribution of the activator element. Relos outputs the expected Poisson distribution for expression. Figure 2b shows the results from an activator-repressor combination. Because expression is now a function of two inputs, it follows the expected Gaussian distribution. Figure 2c shows the results from a synergistic rule set, with noise at 5% of the expression level. In this simulation, each element has a small additive effect on expression individually, but when both regulatory elements are present in the same promoter, a large expression effect is observed. As expected, the result of the simulation is a bimodal distribution, where the second peak represents promoters containing both regulatory elements. Figure 2d shows the output of a cooperative interaction, modeled by a Hill function. A Hill function is a transition function of the form:

$$y=\frac{x^{n}}{\left(\phi^{n}+x^{n}\right)}$$

Where *x *is the input and *ϕ *and *n *are parameters used to adjust the location and steepness of the transition. Hill functions have been used to model biological cooperativity in proteins such as Hemoglobin [16] and in cis-regulatory interactions [17]. In Figure 2d, *x *is the number of cooperative elements, *n *is 3, and *ϕ *is 5. Since the expression is a function of the number of A-elements, and the number of A-elements is distributed according to the Poisson distribution, the expression pattern should be a function of a Poisson distribution. As expected, the simulator output in Figure 2d follows a Poisson distribution with an elongated right tail. This tail represents the high expression of promoters with multiple cooperative sites. See additional file 2 for the rule-sets used to create figure 2.

### Test datasets

The main motivation for creating the simulator was to synthesize expression datasets for which we know the underlying regulatory rules. These datasets will be necessary to compare the accuracy of different methods that infer cis-regulatory rules because there are no experimental datasets for which the true underlying relationships between cis-regulatory sites are known. We therefore created ten test datasets using different rule-sets. The test datasets vary in the number and types of rules and in the complexity of the rule-set. We have made the datasets and rule sets used to generate them (see additional file 1) available in both Relos format and fasta format. We anticipate that the availability of test datasets will allow researchers to evaluate their own methods and compare their methods against commonly used algorithms that deduce regulatory rules from expression data. While the test data we provide will be useful for researchers who want to get started right away testing their rule-finding algorithms, we emphasize that the real power of Relos is the capability it provides to quickly produce custom data sets for algorithm testing. Researchers can now rapidly create their own test datasets to compare the dependency of any method on any particular parameter (number or sites, types of interactions, noisy data).

### Comparison to experimental data

**Figure 3:**
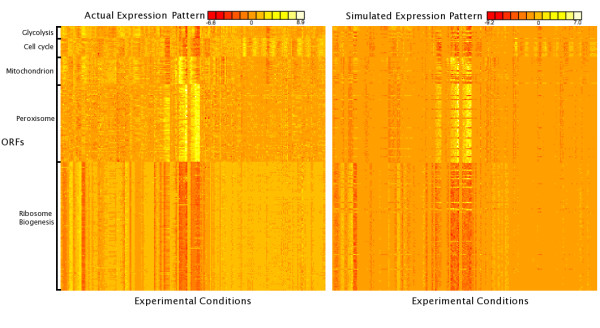
**Comparison of Relos vs. biologically generated data**. Tree regression was performed on five modules (1,11,41,45,49) from Beer and Tavazoie [7]. The tree was converted to a ruleset and the ruleset used to generate expression values for each promoter in the modules. The median gene-wise correlation is 0.78. The real microarray expression values are depicted on the left and the Relos-generated expression values are on the right.

We simulated the expression of five different regulatory modules comprised of 254 yeast genes described in Beer and Tavazoie [7]. A classification tree was constructed to place each gene into its correct module based on the presence or absence of different regulatory elements. Overall, 80% (204/254) of the promoters were placed into their original module. We then created a rule set based on the classification tree which incorporated "AND", "OR", and "NOT" logic. This rule set was used to simulate expression values for each gene in each of the 255 conditions reported in Beer and Tavazoie (see Methods). The results of the simulation and the observed expression values are shown in figure 3. The median gene-wise correlation coefficient between the simulated and experimental expression was 0.78, illustrating that simulated data closely matching observed data can be produced with Relos. These results show that Relos can discriminate between promoters and create biologically relevant data sets.

One noticeable discrepancy between the Relos data and the Beer and Tavazoie data was the noise function. Relos uses Gaussian noise, scaled by the noise-less expression value. This results in a smaller absolute level of noise around expression values close to zero. The Beer and Tavazoie data does not appear to follow this trend; the absolute level of noise around zero is still quite large. Accordingly, we wrote an unscaled noise analyzer that applies unscaled Gaussian noise to simulated data.

We also used the same rule sets defined above to analyze Relos-generated promoters. Randomly generated promoters were created based on the frequency distributions of the cis-regulatory sites that comprised the five modules we simulated. When the rule set was applied to these computationally derived promoters the five expression patterns from Beer and Tavazoie were again recapitulated (see additional file 6). Randomly generated promoters, filtered through Relos, faithfully replicate the observed expression patterns in real data.

## Conclusion

We sought to create a tool that simulates expression from promoters based on cis-regulatory logic. Because there are examples of additivity, synergism, cooperativity, and competition between regulatory sites we created ways to simulate these interactions in a straightforward manner. The full spectrum of interactions between regulatory sites is not known. We recognize that our knowledge of cellular regulation is still relatively limited and that new types of interactions may appear. We therefore did not want to be limited by preconceived models. With its rule-pipeline and analyzer plug-in architecture, Relos allows for virtually any regulatory model to be implemented.

The ease of specifying regulatory models and the speed with which data can be generated will allow algorithms that predict gene expression from promoter sequence to be comprehensively tested. Algorithms that attempt to determine regulatory logic rules from expression and sequence data can be analyzed for their performance with respect to noise, the number of underlying rules, and the complexity of the interactions between the rules. Furthermore, researchers can study the size of the dataset required for an algorithm to recapitulate the rules and the ability of the algorithm to recapitulate the specified rules, as opposed to alternate rule sets which also correlate with the data. We have used Relos to generate a test dataset for use in such studies. We anticipate that the ability to rapidly generate unlimited quantities of simulated expression data will speed the design and comparison of algorithms to decode the cis-regulatory logic that underlies real patterns of gene expression.

The final arbiter of the performance of cis-regulatory rule-finding algorithms will be how well they capture the trends in real data. Algorithms that perform well on synthetic data sets, such as those produced by Relos, will not necessarily perform well on biological data. Because experimentally derived data is still of limited quantity and variable quality, extensive testing on synthetic data is the best way to understand the strengths and limitations of specific rule-finding methods. Testing and training on synthetic data avoids over fitting rule finders on the limited quantities of real data that are now available. Testing rule-finding methods on synthetic data sets will clearly be one of the paths forward on the way to decoding the interactions between cis-regulatory sites.

## Methods

### Promoter generation

Relos generates a promoter as a set of elements. Each promoter element is associated with a "cis-element" and an orientation. Each cis-element has an identifier (eg: A, Oct4, etc), a sequence, and a frequency (expected occurrence). The sequence is only used for output purposes; all built-in rule processing is done on promoter elements.

Relos supports two modes of promoter generation: exact length and expected length. In exact length mode, a cis-element is selected from the user-specified list of elements by a roulette wheel selection process. The selected element is added to the promoter starting from the position furthest upstream of the transcription start site. The element is added in a sense or anti-sense orientation with equal probability. Element selection and addition continues until the number of elements added equals the user-specified length. Relos does not insert spacer elements between cis-elements. Rather, all cis-elements are treated as spacer elements unless a rule is defined which uses the cis-element in a manner inconsistent with a spacer element (see Rule Specification below).

In expected-length mode, the element frequencies are transformed by:

$$D_{i}=\frac{d_{i}}{(1+\frac{1}{E})*\sum_{i=1}^{n}d_{i}}$$

Where *D*_*i *_is the transformed frequency of the *i-*th element, *d*_*i *_is the non-transformed frequency for *i-*th element, *E *is the expected promoter length, and *n *is the number of elements. This results in a distribution of cis-elements that includes a "stop" pseudo-element with probably 1/E. The distribution sums to one and preserves the relative probabilities of the user-specified elements. Promoter elements are added as in the exact length procedure until the stop element is selected.

### Rule specification

Rules are specified in an XML-based format defined by the expression_rules.dtd document type definition file. Each rule is defined in terms of the cis-elements the rule uses, an optional custom analyzer to use in place of the default Relos analyzer, the "output" (the amount by which the rule will affect the current expression level for the promoter), and the "operation" (the way in which the output will affect the current expression). Rules may also define precluded rules. Precluded rules are those that are prevented from operating on a promoter should the precluding rule match. Rules using the default analyzer, or custom analyzers that rely on the default analyzer, may specify one or more conditions that determine whether a particular element on the promoter "matches" the rule. Conversely, these conditions may "exclude" elements on the promoter that should not match the rule.

Conditions are comprised of the cis-element(s) to consider, the allowed position(s) and required orientation of the element(s), and zero or more contexts. Each context defines a cis-element that must appear in the promoter with the element under consideration in the condition. Contexts may include specification of the spacing between the two elements and the orientation of the "context" element.

More details on rule specification can be found in additional file 3.

### Promoter analysis

Relos uses a pipeline to perform rule by rule analysis of the promoters. Typically, promoters are moved through the pipeline in the order in which the rules appear in the simulation XML file. However, when a precluding rule matches a promoter, Relos prevents the precluded rules from operating on the matched promoter. Rules which define a custom analyzer delegate promoter analysis to the custom module. All other rules delegate promoter analysis to the default analyzer. The default analyzer determines the number of elements in a promoter that match the rule and multiplies the number of matches by the output amount to determine the magnitude of the effect on the current promoter expression. Promoter expression is then affected by this amount according to the operation defined for the current rule. Valid operations include add (new expression equals the current expression plus the output); multiply (new expression equals the current expression times the output); exponentiate (new expression equals the old expression raised to the power of the output); and replace (new expression equals the output). Matching is performed on a promoter element-wise basis. If the attributes and contexts of at least one condition and no exclusions match, an element will be considered a match. When no conditions or exclusions are specified, the element only needs to match one of the cis-elements specified by the rule.

Once all promoters have been through the rule pipeline, a user-specified amount of noise is added to each promoter by replacing the current expression value with a random value X, where the probability of replacing the current expression value with X is given by the Gaussian distribution,

$$\text{P}(\text{X})=\frac{1}{\sigma \sqrt{2\pi }}{\text{e}}^{-{\left(\text{X}-\mu \right)}^{2}/(2\sigma^{2})}$$

Where *μ *is the current expression value, *σ *= μ*η, and *η *is the user defined level of noise. The Relos default sets the noise to be 5% of the current expression level.

Relos will also transform the data to fit a sigmoidal curve if specified by the user. For each promoter, the transformed expression value is given by:

$$V_{T}=\frac{1}{1+e^{-(V_{0}-\mu_{T})}}$$

Where *V*_*T *_is the transformed expression value of a particular promoter, *V*_0 _is the original expression for that promoter, and *μ*_*T *_is the mean of the untransformed expression of all promoters in the simulation. An alternative method of transformation is provided by adding a transformation rule with a custom analyzer to the end of the pipeline. Relos provides an example of a transforming analyzer in the SigmoidalTransformAnalyzer, which transforms the data according to:

$$V_{T}=\frac{\varphi }{{\left(1+e^{-\alpha *(V_{0}-\gamma )}\right)}^{\beta }}$$

Where *V*_*T *_is the transformed expression value, *V*_0 _is the original expression, *α *adjusts the slope of the curve at the inflection point, *β *adjusts the position of the inflection point, *γ *determines the expected midline expression, and *ϕ *scales the resulting transformation.

More details on promoter analysis can be found in additional file 3.

### Creating test dataset

Ten test-set simulations were run. Two hundred promoters, comprised of eight cis-elements selected from a pool of four possible elements (A-D), were generated for each simulation, except for test-set simulation ten. A noise level of 5% of the expression level was used. None of the datasets were subjected to upper or lower bound constraints. The first nine test-set simulation rule sets were comprised of: an additive activator, an activator with spacing and ordering constraints, two synergistic rule sets with spacing constraints, two cooperative rule sets, a dominant-negative competitive rule set, a dominant positive rule set, and a rule set with constraints on many elements and an enhancer. In the final test-set simulation, two hundred promoters were generated, each comprised of eight cis-elements selected from a pool of eight possible elements (A-H). The final simulation rule set consisted of multiple additive and non-additive effects, incorporating many of the non-additive effects encountered separately in other rule sets. For more details, see additional file 1.

### Comparison to experimental data

Beer and Tavazoie [7] classified 49 transcriptional modules in *S. cerevisiae*. We simulated the "ribosome biogenesis", "peroxisome", "mitochondrion", "cell cycle", and "glycolysis" modules. These modules were chosen because they vary in size, expression outputs, and regulatory complexity. Promoters with no regulatory motifs were removed from the dataset, leaving 254 promoters. Tree regression [18] was performed to determine the best classification tree for separating the promoters into the five transcriptional modules. Input to the classification for each promoter was their assigned module and the presence or absence of each of the 666 proposed motifs. Based on the structure of the classification tree, a general rule set was constructed (additional file 7). The ruleset was then duplicated for each microarray experiment, except the output for each rule was changed to match the average expression for that module. All 254 promoters were used as input sequences for each of the 255 simulations. Tree regression and statistical calculations were performed in R.

We used Relos to generate synthetic promoters based on the frequency of the motifs used in the above rule set. The frequency of each motif was determined in the 254 biological promoters as the number of times each motif occurred divided by the total number of motifs in these promoters. The frequencies of the remaining biological motifs not considered by the ruleset were conglomerated into a single "Spacer" motif (see additional file 8). Relos was used to generate 1000 promoters which were then analyzed by the same rule set described above, with the addition of an "all spacer" rule.

## Authors' contributions

RDZ designed and wrote the simulator with input from JG. BAC conceived the notion of a promoter-expression simulator. BAC and JG provided guidance during the project. RDZ drafted the manuscript with help from BAC and JG. All authors read and approved the final manuscript.

## Supplementary Material

Additional file 1 Test Datasets. A compressed archive (zip) containing: the rulesets used to generate the test-set datasets (ASCII/xml); the datasets in both Relos and fasta format (ASCII); and histograms of each test-set to provide an overview of the data (PNG). Click here for file

Additional file 2 Figure 2 Rule-sets. A compressed archive (zip) file containing the simulation files (ASCII/xml). used in the generation of figure 2. Click here for file

Additional file 3 Rule Specification and Promoter Analysis. Detailed information on how to specify rules and how promoters are analyzed. Click here for file

Additional file 4 Supplementary Table 1: Relos Dependencies. A listing of modules needed for Relos to run and where they can be obtained. Click here for file

Additional file 5 Relos Source Code. A compressed archive (zip) containing the perl source for running Relos, the xml document-type definitions (DTD) which define simulation files, example simulation files, the README, and the source license (GPL). Click here for file

Additional file 6 Image of modules from generated promoters. A "heat map" image showing the expression from the generated promoters. Promoters with only "Spacer" elements are not depicted. Click here for file

Additional file 7 Sample Ruleset for Biological Comparison. Ruleset used in one of the 255 "microarray" simulations. Click here for file

Additional file 8 Ruleset Used for Promoter Generation. The ruleset used to generate the 1000 promoters used in testing the biological relevance of Relos-generated promoters. Click here for file
