# Supplementary figures and images for: A cis-regulatory logic simulator
Source: BMC Bioinformatics. 2007 Jul 27;8:272. doi: 10.1186/1471-2105-8-272 (PMC2375358; doi:10.1186/1471-2105-8-272)

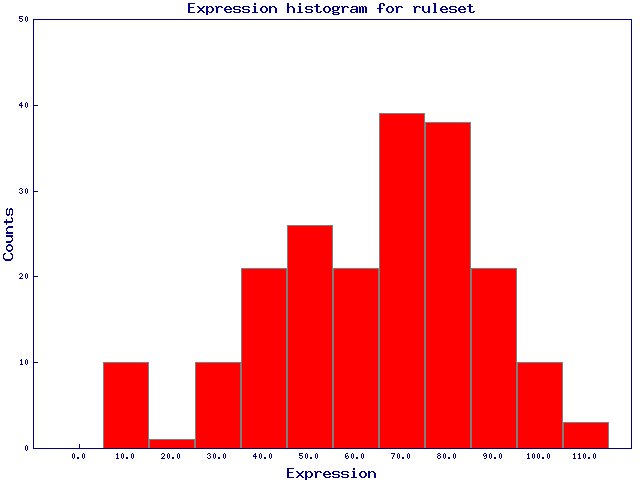

Supplement: Additional file 1 — Test Datasets. A compressed archive (zip) containing: the rulesets used to generate the test-set datasets (ASCII/xml); the datasets in both Relos and fasta format (ASCII); and histograms of each test-set to provide an overview of the data (PNG). [file 1471-2105-8-272-S1.zip › relos/testsets/datasets/distribution10.png]

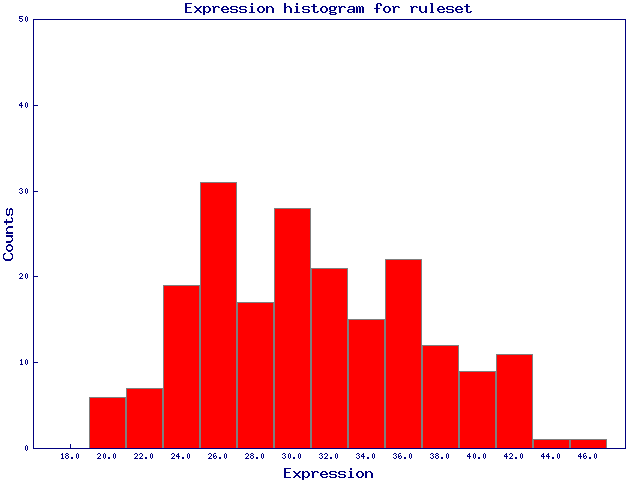

Supplement: Additional file 1 — Test Datasets. A compressed archive (zip) containing: the rulesets used to generate the test-set datasets (ASCII/xml); the datasets in both Relos and fasta format (ASCII); and histograms of each test-set to provide an overview of the data (PNG). [file 1471-2105-8-272-S1.zip › relos/testsets/datasets/distribution1.png]

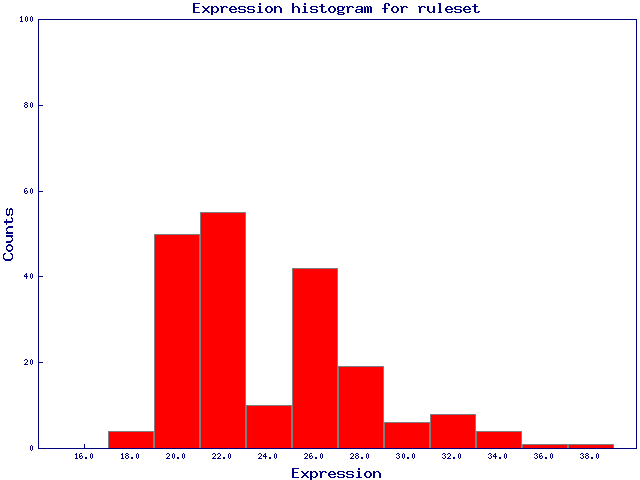

Supplement: Additional file 1 — Test Datasets. A compressed archive (zip) containing: the rulesets used to generate the test-set datasets (ASCII/xml); the datasets in both Relos and fasta format (ASCII); and histograms of each test-set to provide an overview of the data (PNG). [file 1471-2105-8-272-S1.zip › relos/testsets/datasets/distribution2.png]

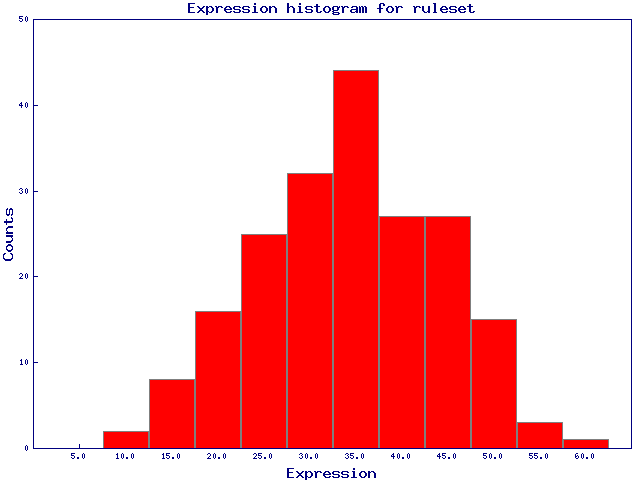

Supplement: Additional file 1 — Test Datasets. A compressed archive (zip) containing: the rulesets used to generate the test-set datasets (ASCII/xml); the datasets in both Relos and fasta format (ASCII); and histograms of each test-set to provide an overview of the data (PNG). [file 1471-2105-8-272-S1.zip › relos/testsets/datasets/distribution3.png]

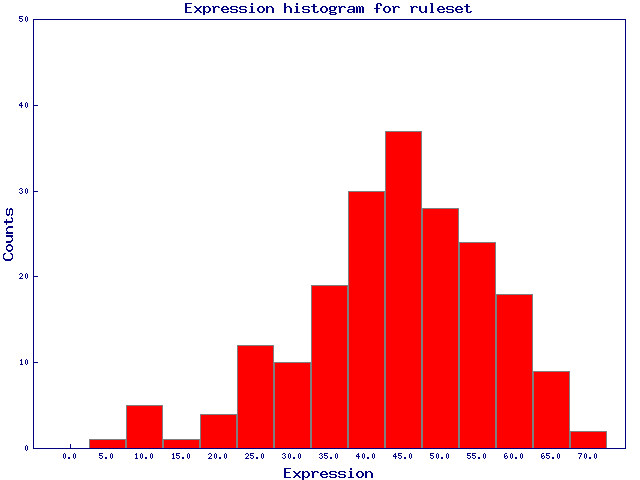

Supplement: Additional file 1 — Test Datasets. A compressed archive (zip) containing: the rulesets used to generate the test-set datasets (ASCII/xml); the datasets in both Relos and fasta format (ASCII); and histograms of each test-set to provide an overview of the data (PNG). [file 1471-2105-8-272-S1.zip › relos/testsets/datasets/distribution4.png]

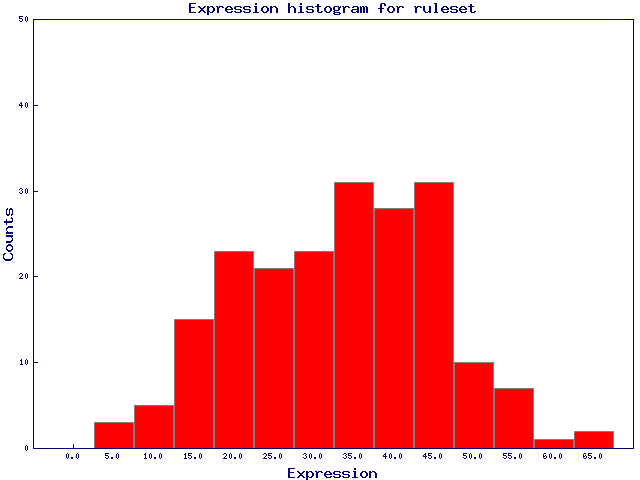

Supplement: Additional file 1 — Test Datasets. A compressed archive (zip) containing: the rulesets used to generate the test-set datasets (ASCII/xml); the datasets in both Relos and fasta format (ASCII); and histograms of each test-set to provide an overview of the data (PNG). [file 1471-2105-8-272-S1.zip › relos/testsets/datasets/distribution5.png]

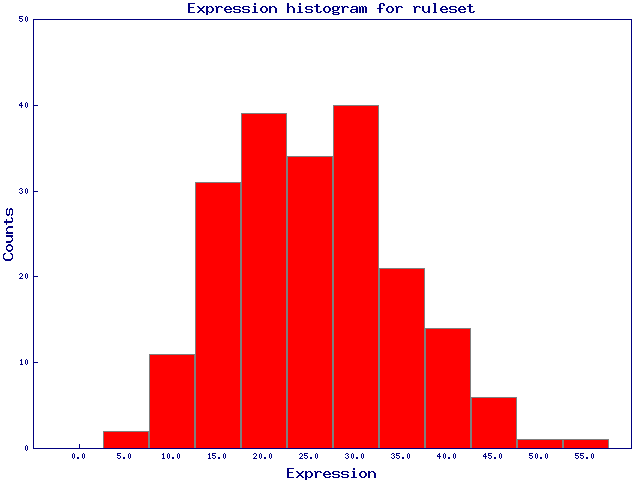

Supplement: Additional file 1 — Test Datasets. A compressed archive (zip) containing: the rulesets used to generate the test-set datasets (ASCII/xml); the datasets in both Relos and fasta format (ASCII); and histograms of each test-set to provide an overview of the data (PNG). [file 1471-2105-8-272-S1.zip › relos/testsets/datasets/distribution6.png]

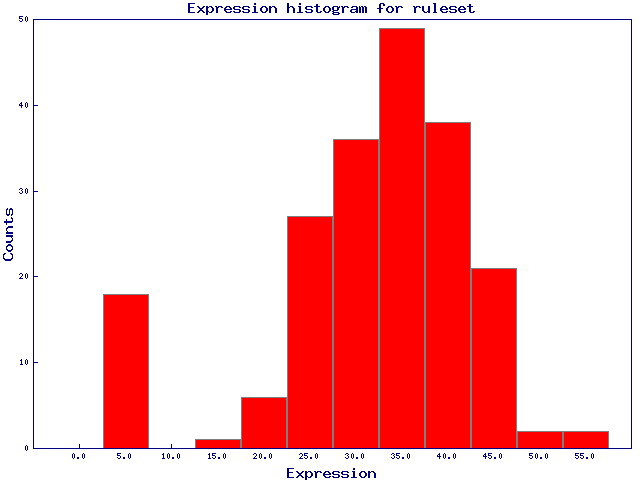

Supplement: Additional file 1 — Test Datasets. A compressed archive (zip) containing: the rulesets used to generate the test-set datasets (ASCII/xml); the datasets in both Relos and fasta format (ASCII); and histograms of each test-set to provide an overview of the data (PNG). [file 1471-2105-8-272-S1.zip › relos/testsets/datasets/distribution7.png]

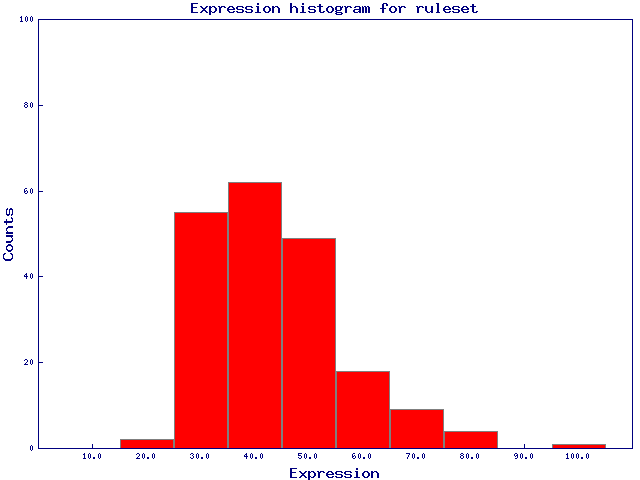

Supplement: Additional file 1 — Test Datasets. A compressed archive (zip) containing: the rulesets used to generate the test-set datasets (ASCII/xml); the datasets in both Relos and fasta format (ASCII); and histograms of each test-set to provide an overview of the data (PNG). [file 1471-2105-8-272-S1.zip › relos/testsets/datasets/distribution8.png]

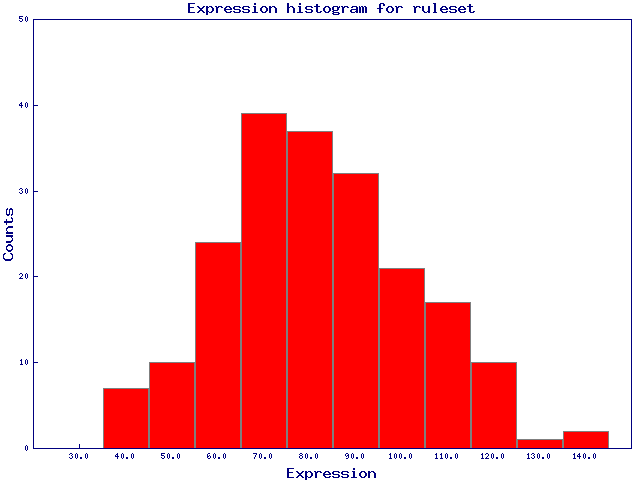

Supplement: Additional file 1 — Test Datasets. A compressed archive (zip) containing: the rulesets used to generate the test-set datasets (ASCII/xml); the datasets in both Relos and fasta format (ASCII); and histograms of each test-set to provide an overview of the data (PNG). [file 1471-2105-8-272-S1.zip › relos/testsets/datasets/distribution9.png]

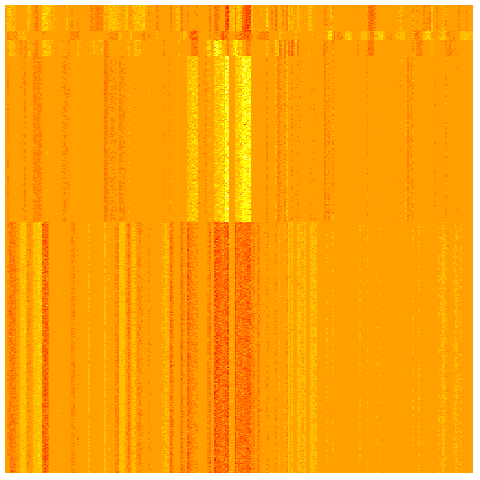

Supplement: Additional file 6 — Image of modules from generated promoters. A "heat map" image showing the expression from the generated promoters. Promoters with only "Spacer" elements are not depicted. [file 1471-2105-8-272-S6.png]
