# Supplementary material for: A cis-regulatory logic simulator
Source: BMC Bioinformatics. 2007 Jul 27;8:272. doi: 10.1186/1471-2105-8-272 (PMC2375358; doi:10.1186/1471-2105-8-272)
Supplement: Additional file 4 — Supplementary Table 1: Relos Dependencies. A listing of modules needed for Relos to run and where they can be obtained. [file 1471-2105-8-272-S4.pdf]

RELOS DEPENDENCIES

The command-line version of relos depends on the following perl modules.

Module	Availability	Optional	Used For
XML::Checker::Parser	http://search.cpan.org/~enno/libxml-enno-1.02/lib/XML/Checker/Parser.pm	NO	Reading rule files
XML::SimpleObject	http://search.cpan.org/~dbrian/XML-SimpleObject-0.53/SimpleObject.pm	NO	Reading rule files
Statistics::Distrib::Normal	Included with Relos source	NO	Noise calculations
GD	http://search.cpan.org/~lds/GD-2.35/GD.pm	YES	PNG histograms
GD::Text	http://search.cpan.org/~mverb/GDTextUtil-0.86/Text.pm	YES	PNG histograms
GD::Graph	http://search.cpan.org/~bwarfield/GDGraph-1.4308/Graph.pm	YES	PNG histograms
Math::Symbolic	http://search.cpan.org/~smueller/Math-Symbolic-0.507/lib/Math/	YES	EquationAnalyzer
